# Integrin α6β4 Identifies Human Distal Lung Epithelial Progenitor Cells with Potential as a Cell-Based Therapy for Cystic Fibrosis Lung Disease

**DOI:** 10.1371/journal.pone.0083624

**Published:** 2013-12-12

**Authors:** Xiaopeng Li, Nathan Rossen, Patrick L. Sinn, Andrew L. Hornick, Benjamin R. Steines, Philip H. Karp, Sarah E. Ernst, Ryan J. Adam, Thomas O. Moninger, Dana N. Levasseur, Joseph Zabner

**Affiliations:** 1 Department of Internal Medicine, Roy J. and Lucille A. Carver College of Medicine, University of Iowa, Iowa City, Iowa, United States of America; 2 Department of Pediatrics, Roy J. and Lucille A. Carver College of Medicine, University of Iowa, Iowa City, Iowa, United States of America; University of Colorado, Denver, United States of America

## Abstract

To develop stem/progenitor cell-based therapy for cystic fibrosis (CF) lung disease, it is first necessary to identify markers of human lung epithelial progenitor/stem cells and to better understand the potential for differentiation into distinct lineages. Here we investigated integrin α6β4 as an epithelial progenitor cell marker in the human distal lung. We identified a subpopulation of α6β4^+^ cells that localized in distal small airways and alveolar walls and were devoid of pro-surfactant protein C expression. The α6β4^+^ epithelial cells demonstrated key properties of stem cells *ex vivo* as compared to α6β4^-^ epithelial cells, including higher colony forming efficiency, expression of stem cell-specific transcription factor Nanog, and the potential to differentiate into multiple distinct lineages including basal and Clara cells. Co-culture of α6β4^+^ epithelial cells with endothelial cells enhanced proliferation. We identified a subset of adeno-associated virus (AAVs) serotypes, AAV2 and AAV8, capable of transducing α6β4^+^ cells. In addition, reconstitution of bronchi epithelial cells from CF patients with only 5% normal α6β4^+^ epithelial cells significantly rescued defects in Cl^-^ transport. Therefore, targeting the α6β4^+^ epithelial population via either gene delivery or progenitor cell-based reconstitution represents a potential new strategy to treat CF lung disease.

## Introduction

Cystic fibrosis (CF), which is caused by loss of cystic fibrosis transmembrane conductance regulator (CFTR), affects multiple organs, though lung disease is the main cause of morbidity and mortality in patients with CF [1]. New therapeutic strategies are urgently needed, and one potential avenue is stem/progenitor cell-based therapy. The long-term vision is to use stem cell-based therapy to regenerate the defective epithelia and thereby reverse the physiological and pathological abnormalities caused by the loss of CFTR. However, these approaches are still in their infancy and require extensive research, including a better understanding of the processes by which stem cells transition to progenitor cells and eventually become differentiated lung epithelial cells.

Use of mesenchymal stem cells has been proven unsuccessful in CF lung disease treatment due to inefficient delivery and engraftment and failure to differentiate to a lung epithelial lineage [2]. Current strategies include the use of induced pluripotent stem (iPS) and embryonic stem (ES) cells or lung-derived adult stem cells/progenitor cells, with each approach having distinct advantages and disadvantages [1]. For iPS and ES cells, the challenge is how to induce selective differentiation to a lung epithelial lineage while avoiding teratoma formation [3]. By contrast, adult stem cells/progenitor cells from the lung represent a potentially safer approach, and these cells are programmed toward a lung epithelia fate [3]. However, the existence of multipotent epithelial stem cells that can give rise to both airway and alveolar epithelial cell lineages in the adult lung is still controversial [3,4]. For example, lineage tracing studies targeting known markers for putative adult lung multipotent stem/progenitor cells have failed to identify such a population under non-pathological conditions in mice [5]. Most studies have been done on mice; however, one group has identified c-kit as a marker for multipotent progenitor cells in the human lung, but confirmative data have not been independently reported by lineage tracing [6]. Recent studies identified integrin α6β4 as a marker for multipotent progenitor cells in the murine distal lung [7,8]. In order to develop epithelial progenitor cell-based therapy for CF, it is first necessary to understand if multipotent epithelial progenitor cells exist or if different regions of the lung contain distinct populations of progenitor cells with limited differentiation potential [9,10]. 

While CF lung disease is considered an airway disease characterized by chronic infection and obstruction of the airway, it has been suggested that the distal lung epithelial cells play a central role in the pathogenesis of CF [11]. The distal lung, which includes the small conducting airway and terminal bronchi, may be the disease initiation site [12]. Our objective was to determine if a multipotent progenitor population exists in the distal portion of human lung that gives rise to both alveolar and airway epithelial cells. Herein we demonstrate that α6β4 can be used as a marker for distal lung epithelial progenitor cells. The α6β4-positive cells undergo clonal expansion and differentiation into basal and Clara epithelial cells. We showed that mixing the α6β4^+^ epithelial population from non-CF donors with bronchial epithelial cells from CF donors rescued the defect in chloride ion transport. Moreover, those α6β4^+^ epithelial cells can be targeted by adeno-associated virus serotypes. Thus, our findings provide fundamental information for future stem/progenitor cell-based therapies for CF lung disease.

## Results

### Isolation and localization of human distal lung epithelial progenitor cells

**Figure 1:**
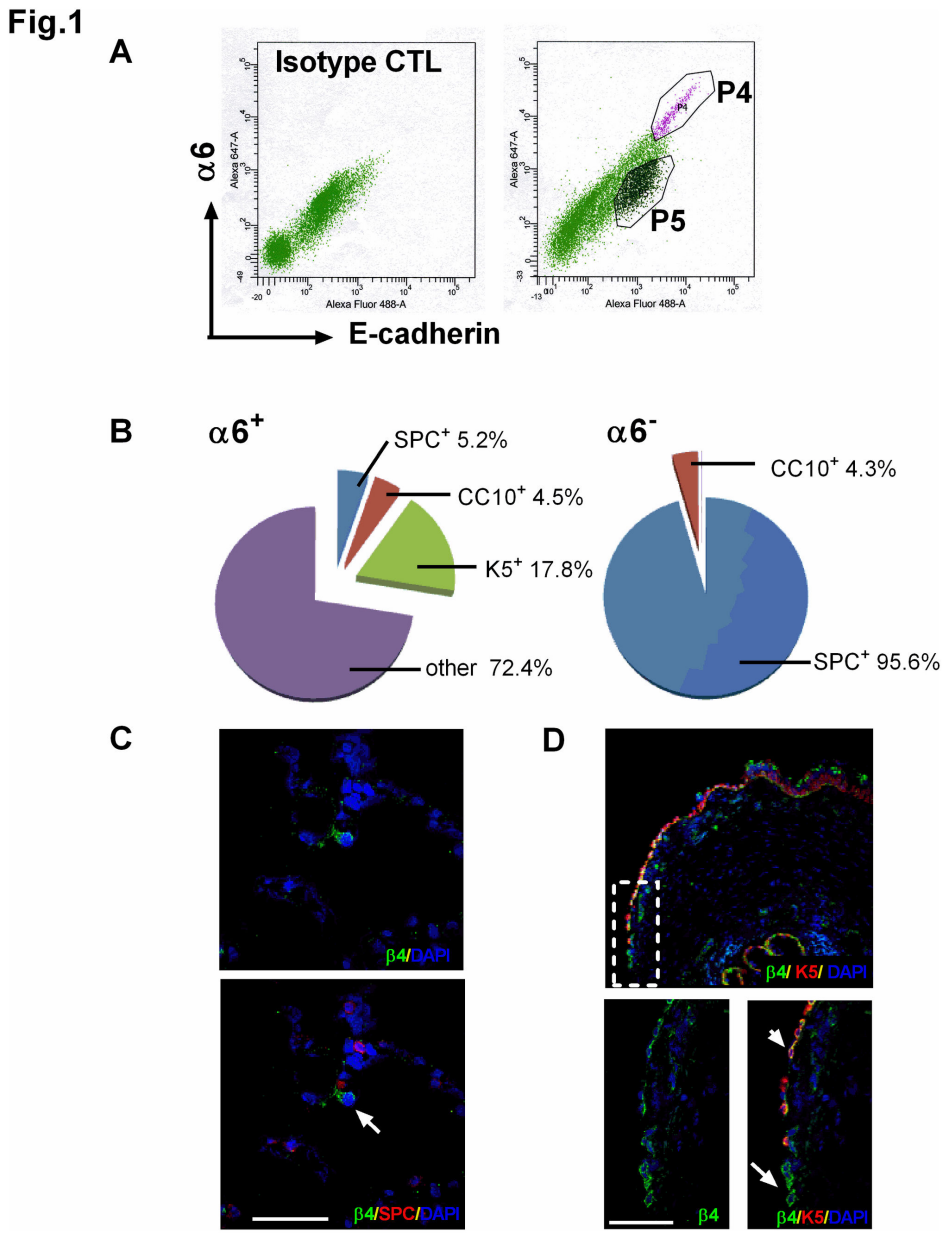
Identification and localization of α6β4^+^ cells in the human distal lung. (A) Epithelial cells were isolated from a normal human lung, and surface markers α6 and E-cadherin were labelled with Alexa Fluor-568 and Alexa Fluor-647, respectively, and analyzed by FACS. Left panel: staining with primary antibody isotype control IgG; right panel: staining with antibodies against α6 and E-cadherin. The P4 gate indicates cells that were positive for both α6 and E-cadherin, whereas the P5 gate indicates E-cadherin^+^ but α6^-^ cells. (B) Populations of cells from the P4 or P5 gate in (A) were isolated, cytospinned on slides, and immediately immunostained with antibodies against SPC, CC10 or K-5. Data are the quantitation of expression of SPC, CC10 and K-5 on either α6^+^ (left pie chart) or α6^-^ (right pie chart) cells. (C, D) Localization of α6β4^+^ cells was determined by co-immunostaining the alveolar (C) region or distal airway (D) in normal human lungs with β4 (green) and SPC (red) or K-5 (red). Nuclei were stained with DAPI (blue). (C) Upper panel, single channel images of β4. Lower panel, merged image of cells co-immunostained with β4 and SPC. Arrow indicates a β4^+^/SPC^-^ cell in the alveolar region. (D) Upper panel, merged image of distal airway at low magnification. Lower panels show enlarged view of dotted box. Lower left panel, single channel images of β4. Lower right panel, merged image of cells co-immunostained with β4 and K-5. Arrow indicates a β4^+^/K-5^-^ cell in the distal airway; arrowhead indicates a β4^+^/K-5^+^ cell in the distal airway. Scale bar= 50 µm.

Given that the data regarding the presence of a multipotent lung epithelial progenitor cell population are conflicting [3,4], our objective in this study was to investigate whether multipotent progenitor cells are present in human distal lungs. The distal lung is defined as the parenchymal lung tissue, including terminal bronchiole and alveolar tissue. In previous murine studies, α6β4 integrin has been identified to be a marker for lung epithelial cells with progenitor potential [7,8]. While integrin α6 has the ability to dimerize with either integrin β1 or β4 [13], integrin α6 predominantly pairs with integrin β4 in murine lungs [8], thus validating the use of a specific α6 antibody. To test if the same marker can be used to distinguish a putative progenitor population in human distal lungs, we isolated human distal lung epithelial cells using a protocol established for isolating type II alveolar epithelial cells [14-16] and examined expression of α6. In the distal human lungs cell isolation, ~4% of cells displayed expression of both the epithelial cell marker E-cadherin (Ecad^+^) and α6 integrin (α6^+^, Figure 1A). Data in Figure S1 in File S1 validate that the α6^+^ cells are also positive for β4 integrin. 

To determine whether the α6^+^ epithelial (Ecad^+^) cells represent a *bona fide* progenitor cell population, expression of markers of airway (basal and Clara) and alveolar (type II) epithelial cells was assessed. Other potential lineages such as hematopoietic, endothelial, and mesenchymal lineages were not queried since cells were gated using the epithelial cell lineage marker Ecad. As shown in Figure 1B, only a small percentage of α6^+^ cells expressed Clara cell secretory protein 10 kDa (CC10), a marker for airway Clara cells (4.5%), keratin-5 (K-5), a marker for basal airway cells (17.8%), or pro-surfactant protein C (SPC), a marker for type II alveolar epithelial cells (5.2%). By contrast, a substantial percentage of α6^-^ epithelial cells contained high expression of SPC (95.6%) but not CC10 (4.3%, Figure 1B), indicating type II alveolar epithelial cell identity as anticipated based on the isolation protocol [14-16]. 

To determine the *in situ* localization of putative progenitor cells, we performed co-staining of α6β4^+^ cells with known markers of other lung epithelial lineages. Immunostaining revealed the *in situ* localization of β4-positive epithelial cells to the alveolar and distal conducting airway regions of the lung (Figure 1C, D) with no co-localization with SPC-positive cells (Figure 1C, lower panel). K-5^+^ cells have a broad distribution within the human airway, with localization from the trachea to the bronchiole-alveolar junction of the distal lung [4], as shown in Figure 1D upper panel. Our data demonstrate that a majority of K-5^+^ cells in the airway were positive for β4 (Figure 1D, upper panel). However, we detected a population of β4^+^ cells that were K-5 negative, located at the distal conducting airway near the bronchiole-alveolar duct junction (Figure 1D, lower panel). Taken together, these data demonstrate that the majority of α6β4^+^ cells from the distal lung have a profile that is distinct from that observed in other well-characterized progenitor cell populations such as basal, Clara, and type II alveolar epithelial cells. 

### Clonal expansion and differentiation of α6β4^+^ epithelial cells

**Figure 2:**
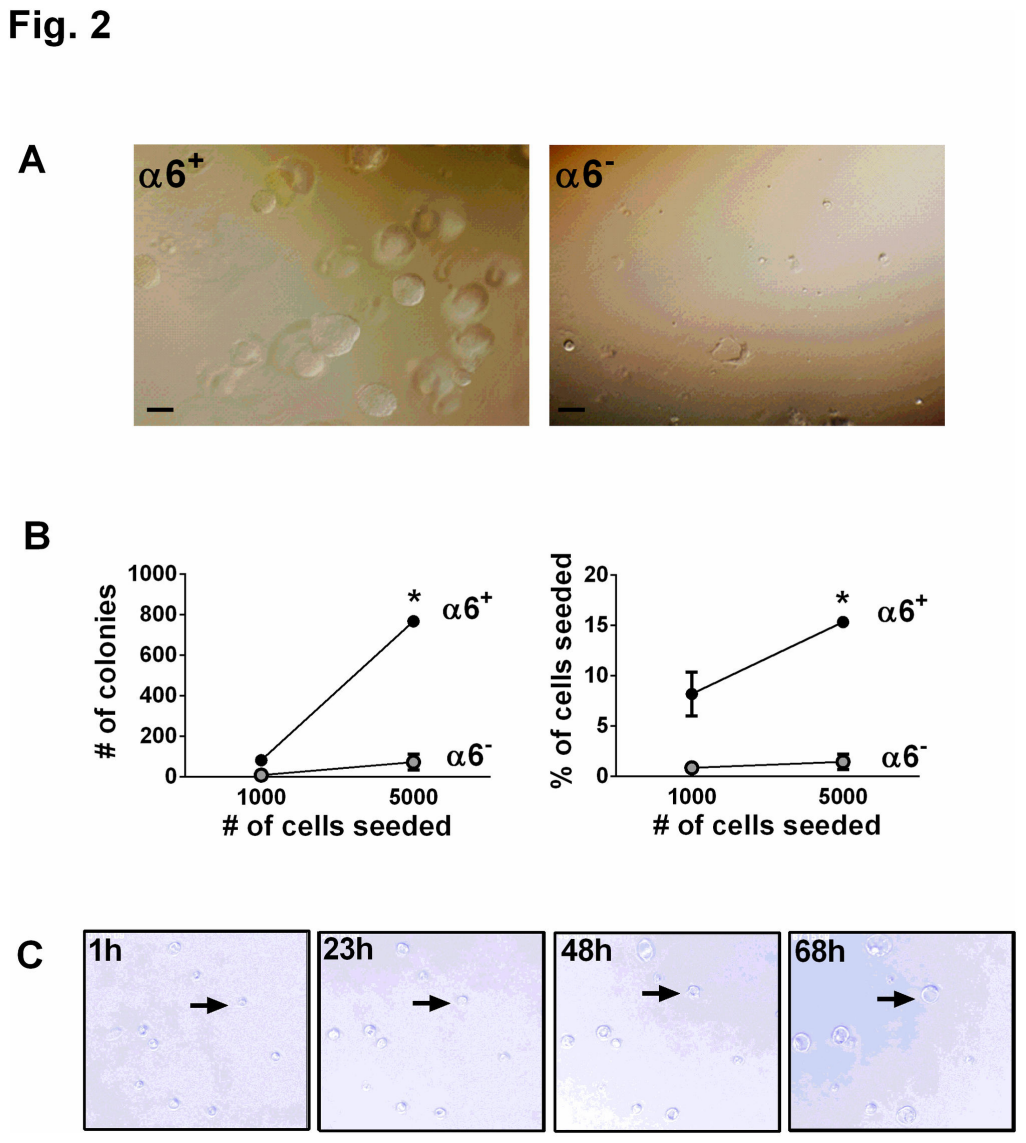
Human α6β4^+^ cells proliferate, undergo clonal expansion, and form colonies from a single cell *ex* *vivo*. (A) Phase contrast images of α6^+^ (left panel) and α6^-^ (right panel) cells from a normal human lung cultured 14 days in Matrigel. Scale bar=200 µm. (B) Colony forming efficiency was analyzed by counting the number of colonies present 14 days after seeding either 1000 (1K) or 5000 (5K) α6^+^ or α6^-^ cells in Matrigel. Left panel, number of colonies; right panel, percentage of cells that gave rise to colonies. Data are shown as mean ± SEM; n=3 experiments using samples from 3 independent donors. * *P*<0.05. (C) Time lapse phase contrast images of colony formation from single freshly-isolated α6^+^ cells. Human α6^+^ cells were isolated from normal human lung and embedded in Matrigel for 2 days, and then time lapse images were taken at the indicated time points. Arrow denotes representative colony formation.

Two defining characteristics of progenitor cells are the capacity for self-renewal (proliferation) and differentiation. Therefore, we first examined clonal expansion of α6^+^ and α6^-^ cells cultured in Matrigel for two weeks. Large clusters indicative of proliferation were apparent in the α6^+^ but not the α6^-^ population (Figure 2A, B). Furthermore, time-lapse videography demonstrated that the α6^+^ colonies originated from a single cell rather than random clustering of cells during plating (single image stills are presented in Figure 2C, see also Video S1), indicative of clonal expansion. The colony-forming efficiency of α6^+^ cells was approximately 10-15% (Figure 2B). By contrast, only 1% of α6^-^ cells formed colonies (Figure 2B). 

**Figure 3:**
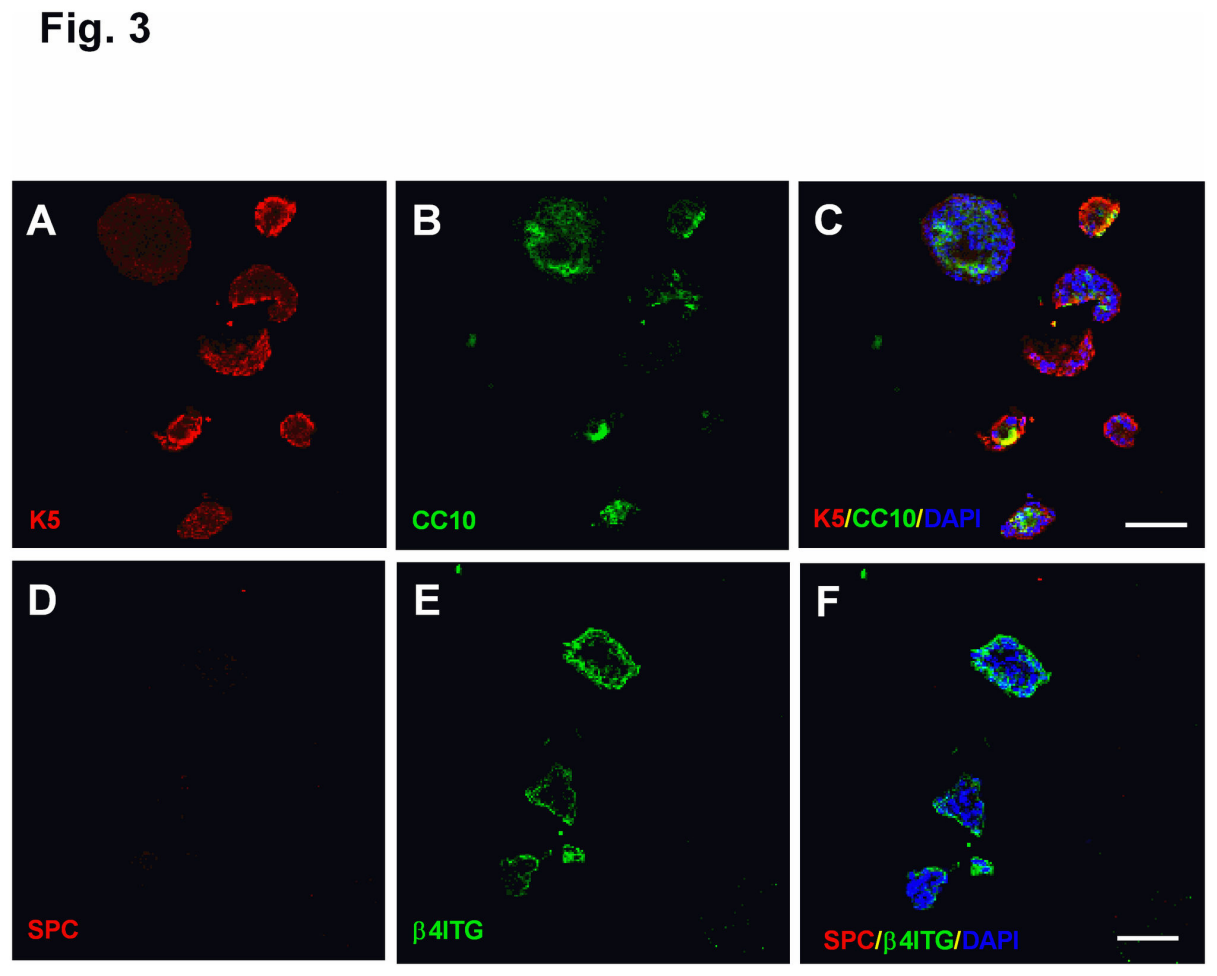
Human α6β4^+^ cells differentiate into both basal and Clara cells *ex* *vivo*. α6^+^ cells were isolated from normal human distal lung and cultured in Matrigel for 33 days. (A, B, C) Colonies were stained with (A) basal cell marker K-5 (red) and (B) Clara cell marker CC10 (green). (C) Merged image of K-5 and CC10. (D, E, F) Immunostaining of colonies with (D) type II AEC marker SPC (red) and (E) β4 (green). (F) Merged image of SPC and β4 staining. Nuclei were stained with DAPI (blue). Scale bar=100 µm.

Next, we examined whether α6β4^+^ cells have the potential to differentiate into different epithelial lineages. The majority of freshly-sorted α6^+^ cells were initially K-5 and CC10 negative. After α6^+^ cells were cultured for 30 days in Matrigel, nearly all of the colonies expressed either K-5 or CC10 or both (Figure 3A-C), which is unlikely entirely due to clonal expansion of the initial population of α6β4^+^ cells that expressed K-5 and CC10. Instead, these data are indicative of differentiation into airway epithelial cells. K-5 expression was limited to the periphery of the colonies (Figure 3A). Interestingly, CC10-positive cells were predominantly located in the center of the colonies (Figure 3B). We previously reported that α6β4^+^ cells isolated from the murine distal lung differentiate into SPC^+^ cells [8]. However, we did not observe SPC expression under the same culture conditions, and the periphery of the colony maintained β4 expression (Figure 3D-F). Semi-quantitative analysis of colonies with different cell type markers demonstrated that 50% of colonies express only K-5, 5% of colonies express CC10 only, and 45% of colonies express both K-5 and CC10. No colonies were detected that express SPC. A positive control for SPC staining of cells is provided in Figure S2 in File S1. Type I alveolar epithelial cell markers T1α and calveolin-1 were not detected under the same culture conditions (data not shown). These data suggest that other factors may be necessary to promote differentiation of human distal lung α6β4^+^ cells into type II alveolar cells. 

**Figure 4:**
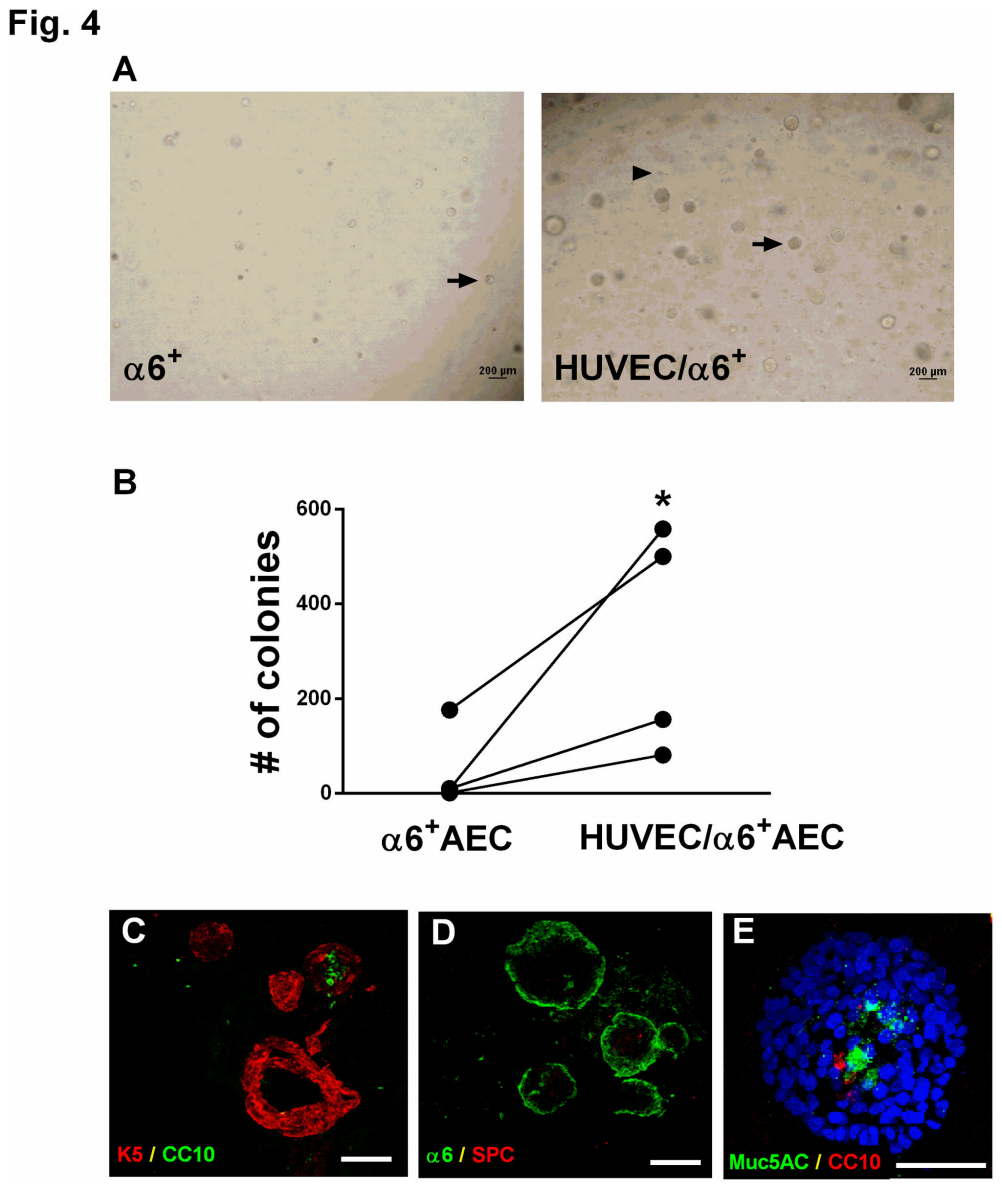
Co-culture of α6β4^+^ cells with HUVECs *ex* *vivo* enhances proliferation but does not alter differentiation. (A, B) α6^+^ cells were isolated from normal human distal lung, mixed with HUVECs, and cultured in Matrigel for two weeks. (A) Phase contrast images of α6^+^ cells cultured without HUVECs (left panel) or with HUVECs (right panel). Arrow indicates colonies arising from α6β4^+^ cells; arrowhead indicates single HUVEC cells. Scale bar= 200 µm. (B) Colony forming efficiency was analyzed by counting the number of colonies present 14 days after seeding 5000 α6^+^ cells in the presence or absence of HUVECs. * *P*<0.05 compared to α6β4^+^ cells alone. Data are representative of 4 experiments from 4 independent donors. Lines denote paired samples from the same donor. (C) Merged image of colonies arisen from co-culture of α6β4^+^ cells and HUVEC stained with K-5 (red) and CC10 (green). Scale bar=100 µm. (D) Merged image of immunostaining with SPC (red) and α6 (green) in colonies arisen from co-culture of α6β4^+^ cells and HUVEC. Scale bar=100 µm. (E) Merged image of immunostaining with goblet cell marker Muc5AC (green) and CC10 (red) in colonies arisen from co-culture of α6β4^+^ cells and HUVEC. Nuclei were stained with DAPI (blue). Scale bar=50 µm.

From a therapeutic perspective, it would be advantageous to accelerate clonal expansion and induce differentiation of α6β4^+^ cells into airway or alveolar epithelial cells *in vitro*. Thus, we next sought to identify ways to promote proliferation and differentiation of α6β4^+^ cells. Others have reported that the interaction between epithelial and endothelial cells is important for lung development [17,18]. In addition, co-culture of immortalized β4^+^ airway basal cells with human vascular endothelial cells (HUVECs) promotes proliferation and differentiation of airway epithelial cells, as evidenced by formation of branched airway structures [19]. When α6^+^ cells were co-cultured with HUVECs, we observed enhanced efficiency of colony formation as compared to cultures of α6^+^ cells alone (Figure 4A, B). Conversely, few colonies formed when α6^-^ cells were co-cultured with HUVECs (data not shown). We did not detect development of bronchi-like structures in the α6^+^/HUVEC co-cultures (Figure 4A). We detected a similar pattern of differentiation when α6^+^ cells were co-cultured with HUVECs as compared to cultures of α6^+^ cells alone (Figure 4C, D). Specifically, a6^+^ cells differentiated into K-5- and CC10-positive (Figure 4C) but not SPC-positive cells (Figure 4D) when co-cultured with HUVECs. In addition, localization of K-5-positive cells to the colony periphery and CC10 to the colony interior was maintained in co-cultures of α6^+^ cells with HUVECs (Figure 4C). We also examined the goblet cell marker Muc5AC and detected expression in the center of the colonies (Figure 4E), further denoting differentiation towards an airway lineage. These data indicate that co-culture of a6β4^+^ epithelial progenitor cells with endothelial cells does not alter cell differentiation potential. 

**Figure 5:**
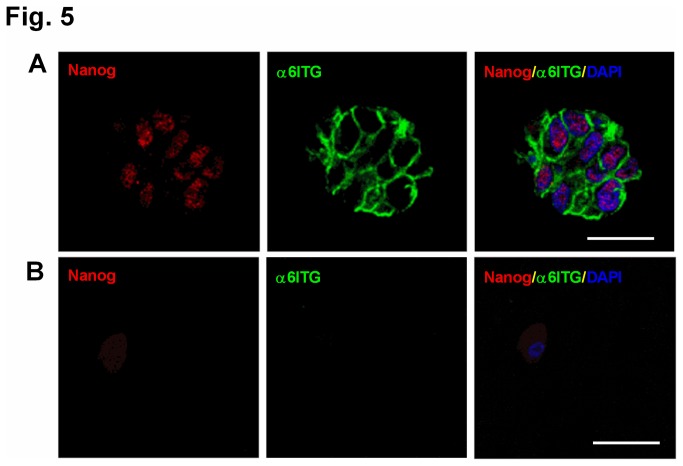
Human α6β4+ cells express stem cell-specific transcription factor Nanog. α6^+^ (A) and α6^-^ (B) cells were isolated from normal human distal lung and cultured in Matrigel for one week, and then colonies were stained with Nanog (red) and α6 (green). Nuclei were stained with DAPI (blue) days. Scale bar=20 µm.

To provide further evidence that α6^+^ cells represent a unique progenitor cell population, we first evaluated expression of the transcription factor Nanog, which has been demonstrated to be expressed in lung stem cells and controls the induction and maintenance of pluripotency in human ES and iPS cells [6,20]. We detected expression of Nanog selectively in the α6^+^ population but not in α6^-^ cells (Figure 5). 

**Figure 6:**
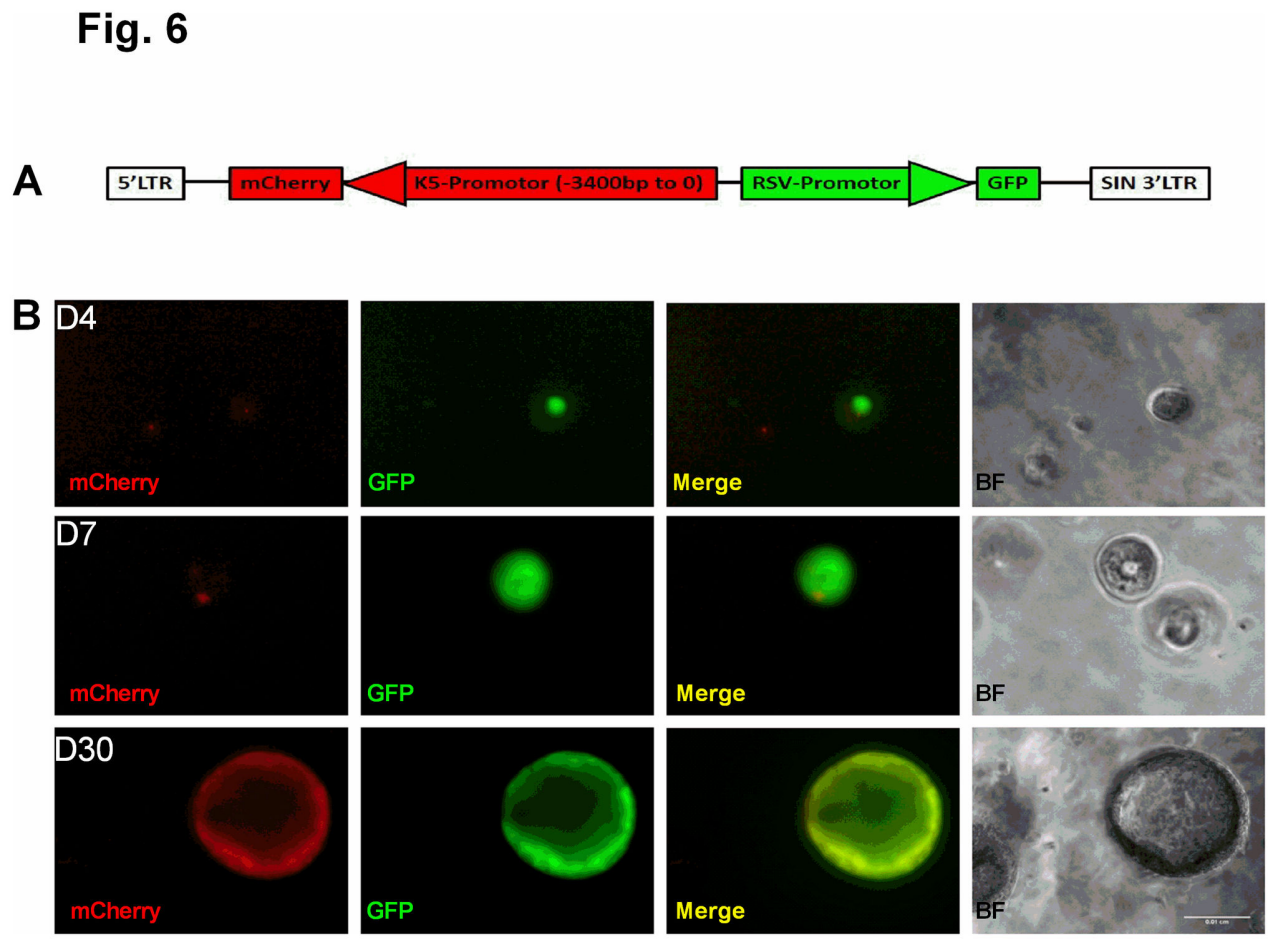
*De* *novo* induction of K-5 expression in human α6^+^ cells. (A) Schematic of dual-color lentiviral reporter construct containing mCherry under the control of a partial K-5 promoter and GFP driven by an RSV promoter. (B) Representative fluorescence images of mCherry (red) and GFP (green) at 4, 7, and 30 days after culture of α6^+^ cells infected with the dual-color lentiviral reporter. Also shown are phase contrast images of colonies. Scale bar=100 µm.

Next, we confirmed that K-5^+^ cells detected in colonies (Figures 3, 4) are differentiated α6^+^ cells rather than expanded K-5^+^ basal cells that were present in the original mixed cell preparation. We constructed a lentiviral reporter vector that contains mCherry driven by a K-5 promoter and GFP driven by an RSV promoter (Figure 6A). Expression of GFP is constitutive, whereas expression of mCherry is indicative of activation of the K-5 promoter. Freshly-sorted α6^+^ cells were infected with the dual-color reporter vector and co-cultured with HUVECs. Representative images in Figure 6B show a time course of GFP and mCherry expression. Whereas GFP expression was detected in colonies early in culture (day 4), mCherry was not detected until day 7, and the intensity was enhanced at day 30 (Figure 6B). Furthermore, expression of mCherry was predominantly in the periphery of the colonies (Figure 6B), consistent with the distribution of K-5 as shown in Figure 3. It is important to note that any mCherry-positive cells observed at day 4 do not undergo clonal expansion like the mCherry-negative/GFP-positive colonies that later become mCherry-positive (Figure S3 in File S1). Taken with the data in Figure 5, these results support that α6 expression is a marker for a unique lung progenitor cell population.

### Approaches to specifically target α6β4^+^ cells

**Figure 7:**
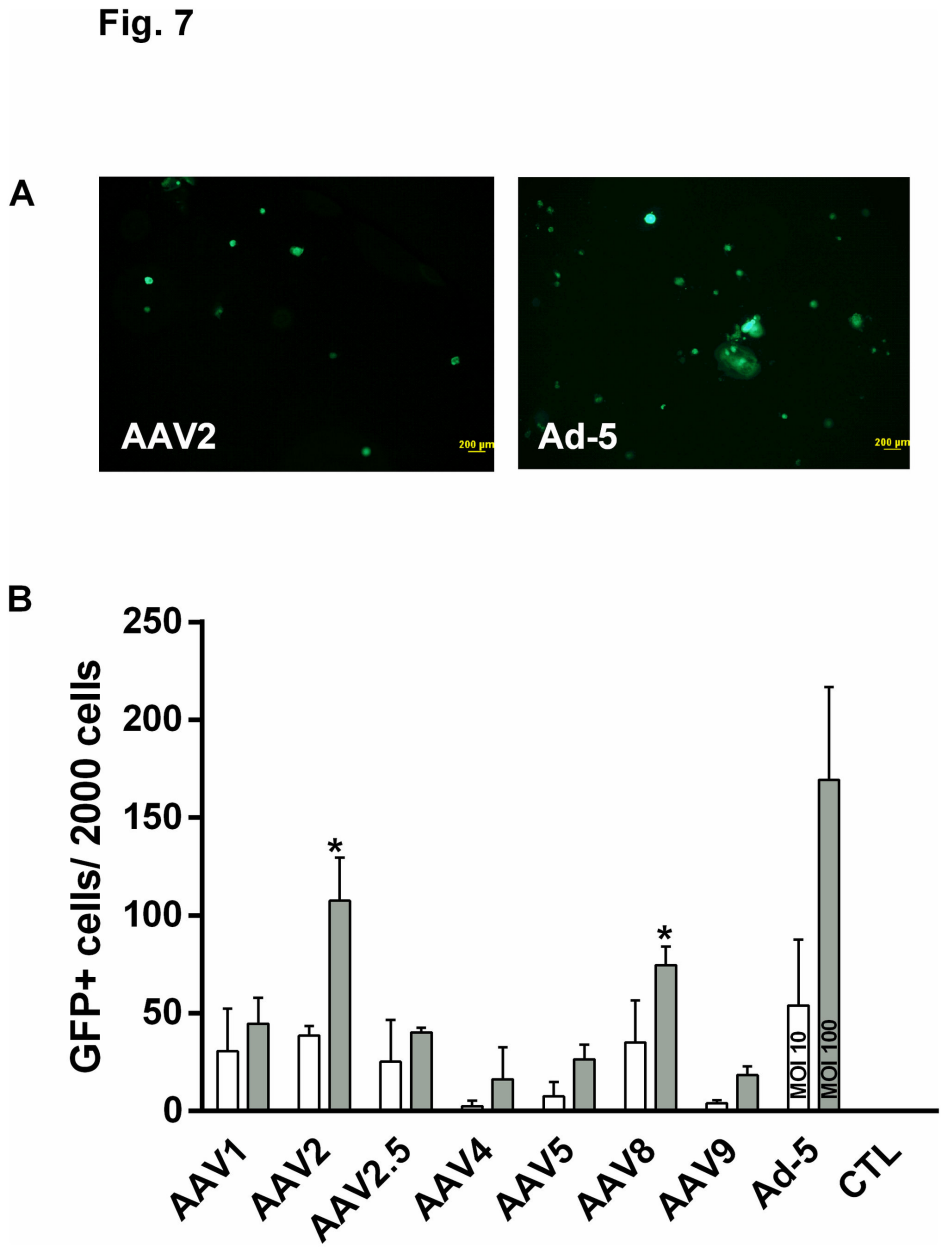
Specific adeno-associated viruses (AAVs) transduce α6β4^+^ cells. A series of AAVs encoding GFP were used to infect α6β4^+^ cells. (A) GFP expression in cells transduced with either 10^7^ AAV2 viral genomes/cell (Vg/cell, left panel) or Ad-5 at an MOI=100 (right panel). (B) GFP^+^ cells/colonies were quantified two weeks after infection with 10^6^ (white bars) or 10^7^ (gray bars) AAV Vg/cell or Ad-5 at MOI=10 (white bar) or MOI=100 (gray bar). CTL: no virus control infection. Data are shown as mean ± SEM; n=3 experiments from 3 independent donors. * *P*<0.05 vs. AAV9 at the same MOI by ANOVA.

Adeno-associated virus (AAV) is an attractive delivery tool for gene therapy approaches due to its non-pathogenic and less immunogenic safety profile, ability to transduce dividing and non-dividing cells, and tissue and species specificity [1,21]. Using AAV to target progenitor cells should allow expression of a therapeutic gene in epithelial cells for a sustained period of time, which has clear advantages for diseases such as cystic fibrosis that are caused by a deficit in a single gene. We conducted a screen to identify which AAV serotype best transduces the α6^+^ cells isolated from the human distal lung. In proof-of-concept studies, a series of AAVs encoding GFP were used to infect α6β4^+^ epithelial cells, with adenovirus-5 (Ad-5) serving as a positive control and no virus as a negative control. The data indicate that AAV2 and AAV8 transduced α6^+^ cells more efficiently than other serotypes as determined by the number of GFP-expressing cells or colonies (Figure 7). Thus, gene transfer to α6β4^+^ cells can be achieved with AAV2 and AAV8. 

**Figure 8:**
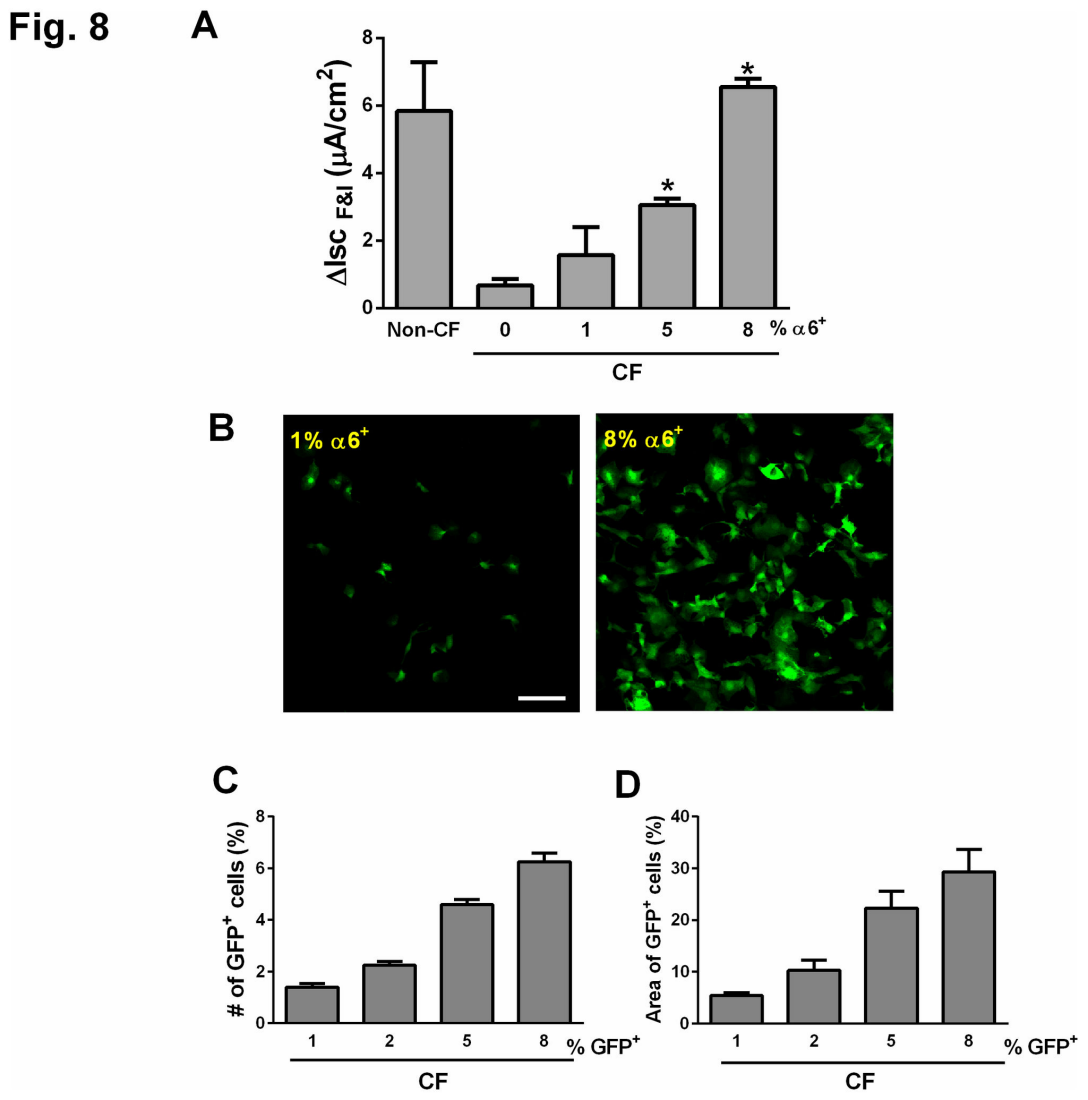
Reconstitution with only 8% of normal human α6β4^+^ cells rescues CFTR function in epithelia from patients with CF. (A) Wild type α6β4^+^ epithelial progenitor cells at various percentages (1%-8%) were mixed with bronchial epithelial cells from patients with CF (CF) and cultured for two weeks in transwells at the air-liquid interface. Responses to cAMP agonists (forskolin + IBMX) were determined by Ussing chamber. Non-CF: bronchial epithelial cells from non-CF lung. Data are shown as mean ± SEM; n=4-5 experiments from 4-5 different donors. *P<0.05 compared to CF 0%. (B-D) Normal human GFP^+^ cells derived from α6β4^+^ epithelial cells were mixed with CF bronchial epithelial cells and cultured for two weeks in transwells at the air-liquid interface. Scale bar=100 µm. (B) Representative enface confocal images of GFP^+^ cells in co-culture with CF bronchial epithelial cells. Scale bar=100 μm. (C) Percent (%) of GFP-positive cells was quantified by flow cytometry. (D) Quantitation of the surface area containing GFP^+^ cells. For (C and D), data are shown as mean ± SEM; n=3 random fields per sample in three independent experiments in D (single donor).

Another potential therapeutic extension of these studies is stem cell-based therapy to replace abnormal epithelial cells with defective CFTR function with cells that express functional CFTR. A defining characteristic of CFTR-deficient epithelial cells is dysregulation of Cl^-^ transport. It has been established that mixing 20-25% wild-type airway epithelial cells with epithelial cells from CF patients can restore CFTR-mediated Cl^-^ current [22,23]. Our goal was to determine 1) whether normal human progenitor α6β4^+^ epithelial cells can also rescue the phenotype associated with CFTR deficiency; and 2) the minimal percentage of human α6β4^+^ cells necessary to restore Cl^-^ current in a population of airway epithelial cells from CF patients. The approach was to mix wild type α6^+^ epithelial progenitor cells at various percentages (1%-8%) with bronchial epithelial cells from patients with CF, and then study CFTR-mediated current in response to agents that increase cAMP levels (forskolin+IBMX). As expected, epithelia from CF patients had a blunted response to forskolin+IBMX as compared to epithelia from non-CF donors (Figure 8A). When increasing percentages of α6^+^ progenitor cells cultured with epithelia from CF patients, we observed a progressive increase in the cAMP-stimulated Cl^-^ current (Figure 8A). Importantly, reconstitution with 5% α6^+^ normal progenitor cells significantly increased cAMP-stimulated Cl^-^ current, and 8% of α6^+^ cells was sufficient to restore Cl^-^ current to levels equivalent to those observed with wild type bronchial epithelia. 

We next examined how a small percentage of α6β4^+^ progenitor cells effectively rescued Cl- current. Freshly sorted α6^+^ cells were infected with a lentivirus containing GFP, and α6^+^/GFP^+^ cells were recovered after one week amplification using recently published protocol [30]. These α6^+^/GFP^+^ cells were then mixed with bronchial epithelial cells from a CFTR null patient and co-cultured for two weeks. Surprisingly, we did not detect an increased percentage of GFP^+^ cells after two weeks in co-culture with CFTR null epithelial (Figure 8B-C), yet we did observe a dramatic increase in the surface area containing GFP^+^ cells (Figure 8D). These data suggest that, due to the disproportionate increase in the surface area of the α6β4^+^-derived population, only a small proportion of α6β4^+^ progenitor cells are necessary to rescue the defect in Cl^-^ transport in epithelia from CF patients.

## Discussion

To realize the full potential of stem cell therapy for diseases such as CF, it is necessary to determine biomarkers that identify progenitor cells in distinct regions of the lung and to understand the potential of these progenitor cells to differentiate into different epithelial cell lineages. In this study we identified a multipotent epithelial progenitor population within the distal human lung based on cell-surface expression of integrin α6β4. As evidence for progenitor cell identity, α6^+^ epithelial cells displayed a higher capacity for self-renewal than α6^-^ cells and the ability to differentiate into basal and Clara cells and goblets. The efficiency of clonal expansion of α6^+^ cells was enhanced by co-culture with endothelial cells. In proof-of-concept gene delivery studies, AAV2 and AAV8 transduced α6^+^ cells with a higher degree of efficiency than other serotypes, suggesting that these serotypes may represent a gene delivery tool for the distal lung epithelial progenitor cell population. As a first step towards lung stem cell-based therapy, we found that mixing the α6^+^ population from normal donors at only 5% with bronchial epithelial cells from CF donors significantly rescued the phenotype associated with defective CFTR function. Identifying human distal lung epithelial progenitor cells may provide unique opportunities, including gene delivery and stem cell replacement therapy, both to study the pathogenesis and to develop new treatment strategies for CF and other lung diseases. 

To date, only a handful of markers, including α6β4, c-Kit, and keratin-5, has been identified as markers of lung progenitor cells [3,6-8,24]. Recently it has been reported that, in murine distal lungs, integrin α6β4 expression identifies a population of cells with multipotent stem/progenitor potential [7,8]. About 10% of cells isolated from the distal murine lung epithelia were high in α6β4 and low in pro-surfactant protein C (SPC) by flow cytometry [7,8], consistent with our data in epithelial cells isolated from the human distal lung in which ~4% of cells were α6-positive. Moreover, the murine subpopulation can differentiate into multiple distinct lineages *in vivo*, including SPC^+^ (type II alveolar epithelial cell marker) cells, CC10^+^ (airway epithelial cell marker), and T1α^+^ (type I alveolar epithelial cell marker) cells [7,8]. Our data show that human α6^+^ cells isolated from the distal lung of normal donors also differentiate into multiple lineages, including CC10^+^ Clara, K-5^+^ basal airway, and Muc5AC^+^ goblet cells, though we did not detect differentiation into SPC^+^ type II alveolar lineages *in vitro*. Recently, it has been reported that after influenza-induced lung injury, a population of K-5 positive cells is present that can differentiate into both airway and alveolar lineages but not SPC^+^ cells *in vivo* [24], providing further support for the hypothesis that a progenitor population exists that can differentiate into multiple epithelial lineages. 

Differentiation is likely highly dependent on the precise environment as demonstrated by the above study in which distal lung-derived progenitor cells can differentiate into an alveolar lineage but do not express SPC [24]. Our data do not exclude the possibility that α6^+^ cells can differentiate into alveolar lineages given the proper environmental signals. It is important to note that the differentiation experiments in the murine studies were conducted *in vivo* [8], which contains a host of factors that may be required for differentiation into the alveolar lineage. Such *in vivo* studies are technically challenging in our model since lineage tracing is required for *in vivo* studies, and we utilized cells of human origin.

To rule out the possibility that we isolated another known progenitor population in the distal lung such as basal or Clara cells that give rise to goblet and ciliated cells, we stained α6^+^ cells for markers of basal, Clara and type II alveolar cells. A majority of α6^+^ cells (72.4%) did not express markers of Clara or basal cells or mature type II alveolar epithelial cells, suggesting a novel progenitor population. One potential explanation for the higher percentage of α6^+^/K-5^+^ cells (17.8%) relative to our previous study in mice [8] is that, in humans, basal cells rather than Clara cells are distributed throughout the airway, from the trachea to the bronchiole-alveolar junction area. In previous work, another marker, nerve growth factor receptor (NGFR), in addition to α6 was used to isolate basal cells from the proximal airway [25]. However, α6+/NGFR^-^ cells also form colonies in culture [25], precluding use of this marker in our study. A recently published parallel study identified another marker for lung progenitor cells [26], which could be useful to distinguish between cells from the alveolar and distal airway regions. 

It remains possible that we isolated a subpopulation of basal progenitor cells that can only differentiate into airway lineages, but this is unlikely given that 1) after one week in culture, α6^+^ cells expressed the stem cell-specific transcription factor Nanog, and Nanog has not been reported to be expressed in basal or Clara cells; and 2) induction of the K-5 promoter was not apparent until day 7 and increased with prolonged culture. Nevertheless, this population of α6^+^ cells is distinct as compared to previous studies using α6β4^+^ basal (K-5^+^) progenitor cells isolated from the proximal airway/trachea [25]. First, the α6^+^ cells we isolated from the human distal lung formed solid masses rather than cysts with a lumen lined with differentiated ciliated cells [19,25]. Cysts are typically derived from basal cells. Second, unlike co-cultures of β4^+^ basal and endothelial cells [19], we did not detect branching structures when primary α6^+^ cells were co-cultured with endothelial cells *in vitro*, which supports the notion that α6^+^ cells isolated from the distal lung are not basal cells. Taken together, these data suggest that the α6^+^ cells isolated from the distal lung are unique from other described lung epithelial progenitor populations. 

Most reports on lung progenitor cells have been limited to characterization of their ability to differentiate and self-renew. Our study also examined their therapeutic potential *in vitro*. We focused on CF for several reasons. First, the current understanding regarding CF pathogenesis suggests that the disease initiation site is the distal lung [12]. Second, CF is a genetic disease with a single gene mutation that results in loss of function, thus making CF ideally suited for gene therapy or stem cell-based therapy. Furthermore, heterozygotes are asymptomatic, signifying that only one functional CFTR allele is sufficient to prevent disease development. Several studies have suggested that restoring CFTR function by gene therapy or stem cell replacement are attractive strategies to reverse the disease phenotype [1], though none of these approaches have been established as standard clinical use. Herein, we conducted studies to determine whether the α6^+^ cells possess therapeutic value in the setting of CF. Strikingly, we observed that addition of only 8% of WT α6^+^ cells to airway epithelial cells from CF patients was sufficient to restore Cl^-^ current, with a graded effect at lower percentages of WT α6^+^ cells. These data are in contrast to a previous report that observed that 20-25% of airway epithelial cells are needed to rescue the CFTR-mediated phenotype [22,23]. One potential explanation for why we achieved an effect with a much lower percentage of progenitor cells is that, after two weeks of culture with CF cells, the surface area covered by the progenitor-derived population was significantly increased. 

In summary, we identify a novel population of cells in the human distal lung with the capacity for self-renewal, clonal expansion, and differentiation into distinct epithelial cell lineages. Our study adds to the literature regarding the existence of adult lung progenitor cell populations by defining expression of α6β4 integrin as a marker of progenitor cells in the distal lung. However, several questions remain. One urgent need is to understand whether various lung diseases alter the properties of this population of progenitor cells. We must also determine whether human α6β4^+^ epithelial cells can mediate repair of lung tissue following injury. Finally, the full potential of this population to differentiate into multiple epithelial cell lineages remains unknown. Despite these unanswered questions, recognizing the presence of such a population in the distal lung has important implications in future efforts for targeted treatment of a wide scope of lung diseases, including cystic fibrosis, pulmonary fibrosis, surfactant deficiency, and lung cancer. These studies may require decades of research to identify the best strategy to deliver progenitor cells to the appropriate region of the lung. A more realistic approach would be to target endogenous progenitor cells using viral vectors evolved to specifically transduce the progenitor cells. 

## Materials and Methods

### Human distal lung cell preparations and flow cytometry to isolate α6β4^+^ epithelial cells

All human lung tissues were obtained from the University of Iowa Cell Culture Core Facility, which acquired tissue from donors under an organ research donation protocol that was approved by the Institutional Review Board of the University of Iowa. Distal human lung epithelial cells were isolated using a method established for isolation of type II alveolar epithelial cells from the distal lung as previously described [14-16]. Briefly, the pulmonary artery was perfused with PBS solution and the distal air spaces were lavaged 10 times with Ca^2+^- and Mg^2+^-free PBS solution (0.5 mM EGTA and 0.5 mM EDTA). A trypsin-elastase combination (0.5 mg/ml elastase in 0.5% trypsin solution) was instilled in the right middle lobe of lung and used to enzymatically digest the distal lung tissue at 37°C for 60 min with shaking. Differential adherence on plastic surfaces served to remove macrophages (incubation at 37°C for 90 min), and dissociation from blood cells and cell debris was performed using a discontinuous Percoll density gradient (p = 1.089 g/ml and p = 1.040 g/ml) and centrifuged at 600 x *g* for 20 min. Cells collected from the interface were used for further analysis. Cells were plated on Matrigel (BD Biosciences, San Jose, CA)-coated plates for two days. Adherent cells were detached by trypsinization, washed, and co-stained with a rat monoclonal α6 antibody (BD Biosciences) and a mouse monoclonal primary antibody against E-cadherin (Lifespan Biosciences, Seattle, WA) followed by secondary antibodies anti-rat Alexa-Fluor 647 and anti-mouse Alexa-Fluor 488 (Invitrogen, Grand Island, NY). Cells were sorted for α6 and E-cadherin expression using the FACSAria III Cell Sorter (BD Biosciences) at the University of Iowa Flow Cytometry Core Facility. 

### Identification of α6β4^+^ epithelial cells

Human lung sections were fixed with 4% paraformaldehyde for 15 min, washed extensively with PBS, and permeabilized with 0.2% Triton X-100. Nonspecific binding was blocked by 1 hr incubation in SuperBlock Blocking Buffer (Pierce, Rockford, IL) and sections were incubated with primary antibodies overnight at 4°C. Primary antibodies used were as follows: 1) mouse anti-β4 (1:50; R&D Systems, Minneapolis, MN); 2) rabbit anti-proSPC (1:100; Invitrogen); and 3) mouse anti-E-cadherin (1:100; Lifespan Biosciences). The following day, sections were washed with SuperBlock plus 2% BSA and incubated with secondary antibodies (goat-anti-rabbit-Alexa-Fluor-568, goat anti-rat-Alexa-Fluor-488, 1:200 in SuperBlock plus 2% BSA; Invitrogen) for 1 h at room temperature protected from light. Following extensive washes with SuperBlock plus 2% BSA, sections were counterstained with DAPI, and inserts were then mounted onto glass slides and coverslipped using Vectashield mounting medium (Vector Laboratories, Burlingame, CA). Images were acquired with identical parameters on an Olympus Fluoview FV1000 confocal microscope as previously described [27]. 

### Clonal expansion and differentiation studies

Sorted E-cadherin-positive epithelial cells (α6^+^ and α6^-^) were seeded in Matrigel at a 1:1 (vol/vol) ratio in transwell filters (Corning, Corning, NY) with a 0.4 µm pore size in a 24-well plate. Cells were maintained in Small Airway Growth Medium (Clonetics, Basel, Switzerland) without hydrocortisone containing 5% charcoal/dextran treated FBS and 10 ng/ml KGF in a 37°C, 5% CO2; media was changed every three days. For clonal expansion studies, colonies were counted after two weeks. Time-lapse images were acquired every 10 min for three days through the University of Iowa Central Microscopy Core Facility; movie file (Video S1) can be viewed with QuickTime Player. For analysis of Nanog expression after one week of culture in Matrigel, transwells were fixed, embedded in OCT, and frozen 10 µm sections cut, followed by staining with Nanog (1:50, Cosmobio, Tokyo, Japan) and α6 (1:100, BD Biosciences). For differentiation studies, sections were obtained 30 days after culture in Matrigel and stained with differentiation markers SPC (1:100, Invitrogen), CC10 (1:100, APC Biotechnology Service, Rockville, MD), keratin-5 (K-5, 1:500, Covance, Princeton, NJ), and mucin 5AC (Muc5AC, 1:50, AbCam, Cambridge, MA) as well as β4 (1:00, BD Biosciences) as described for α6β4^+^ cell identification. Nuclei were counterstained with DAPI and images acquired on an Olympus Fluoview FV1000 confocal microscope. 

### Co-culture of α6^+^ cells with HUVECs

For co-cultures, either 1000 or 5000 α6^+^ cells were mixed with 50,000 HUVECs (gift provided by Drs. Theresa Gioannini and Joseph Dillon at University of Iowa [28]) in Matrigel as described for clonal expansion and differentiation studies [19]. Media was replaced every three days, and images acquired after two weeks in culture as in clonal expansion studies. 

### Dual-color lentiviral reporter assay for de novo K-5 induction

To generate a dual-color lentiviral reporter, we first cloned a portion of the full-length K-5 promoter (nt -3400-0; plasmid generously provided by Dr. Elaine Fuchs at Columbia University [29]) into a lentiviral construct upstream of mCherry (Figure 6A). This FIV vector (obtained from the University of Iowa Gene Transfer Vector Core Facility) also contains GFP downstream of an RSV promoter. Freshly-sorted α6^+^/E-cad^+^ epithelial cells (10,000 cells) were infected at an MOI of 100 and seeded in Matrigel at a 1:1 (vol/vol) ratio in the inner chamber of transwell filters (Corning) with a 0.4 µm pore size. HUVECs (50,000 cells) were seeded in the outer chamber. Cells were maintained in Small Airway Growth Medium and images acquired with a fluorescent inverted microscope on days 4, 7, and 30 after seeding. 

### Adeno-associated virus infection

Adeno-associated viral (AAV) vectors and adenovirus-5 (Ad-5) encoding GFP were obtained from the University of Iowa Gene Transfer Vector Core Facility. Approximately 2000 freshly-sorted α6^+^/E-cad^+^ epithelial cells were inoculated with 10^6^ or 10^7^ viral genomes per cell (vg/cell) of indicated AAV serotypes diluted in EMEM for 2 hrs at 37°C. Ad-5 encoding GFP was used as a positive control at an MOI of 10 and 100 and no virus treatment served as a negative control. Cells were centrifuged at 600 x *g* and washed with EMEM, and then seeded in 50% Matrigel. Transduction efficiency was analyzed by monitoring GFP-positive cells daily by fluorescence microscopy. After one week in culture, 5 µM Hoechst-33342 (prod #H1399, Invitrogen) was used to enhance AAV infection as previously described [30]. 

### Mixed cultures with bronchi from patients with CF

Bronchial cells from non-CF donors and patients with CF were obtained from the University of Iowa Cell Culture Core Facility. α6^+^ cells were removed by FACS from the population of bronchial cells from patients with CF. Freshly-sorted α6^+^ cells from human distal lung were mixed with different proportions of CF α6^-^ bronchial cells. A total of 300,000 cells were seeded onto collagen-coated, semi-permeable membranes and grown as previously described [31] for two days in submerged condition using USG media [32] supplemented with gentamicin, ampicillin (50 mg/ml each) and penicillin G (200 U/ml). From day 3 after initial seeding, cells were cultured at the at the air-liquid interface (ALI) at 37°C in a 5% CO_2_ atmosphere for 2 weeks and the culture medium was replaced at least every two days as described [32]. Bronchial cells from non-CF donors (300,000 cells) were used as a control and plated and cultured under the same protocol.

To visualize α6^+^ cells isolated from the normal human distal lung in the co-cultures with CF α6^-^ bronchial cells, freshly isolated normal α6^+^ cells were infected with a lentivirus containing GFP cDNA (University of Iowa Gene Transfer Vector Core Facility) then cultured for one week to amplify the GFP-positive population as described previously [33]. Briefly, cells were co-cultured with NIH3T3 cells J2 strain (obtained from University of Iowa Cell Culture Core) with 10 µM ROCK inhibitor Y-27632 (Enzo Life Sciences, Switzerland). For this experiment, bronchial epithelial cells from a CF patient with a null mutation of CFTR (CFTR Q493X/S912X, obtained from University of Iowa Cell Culture Core) were amplified for one week using the same method. GFP-positive α6^+^-derived cells were isolated and recovered using the FACSAria III Cell Sorter (BD Biosciences) at the University of Iowa Flow Cytometry Core Facility. These sorted GFP^+^/α6^+^ cells from normal human distal lung were immediately mixed with different proportions of CFTR-null bronchial epithelial cells and cultured as described [32]. After 2 weeks, images were acquired on an Olympus Fluoview FV1000 confocal microscope after nuclear staining with DAPI. GFP-positive cells were quantified by counting the number of GFP-positive cells by flow cytometry and by determine the percent surface area covered by GFP-positive cells using NIH image J software. 

### Ussing chamber studies

Ussing chamber studies were performed on mixed cultures of α6^+^ and CF bronchial cells, bronchi from non-CF donors, or CF bronchial cells alone two weeks after seeding. Cells were mounted in Ussing chambers and studied as previously described [34-36]. Apical and basolateral chambers contained the same bathing solution with symmetrical Cl^-^ concentrations. CFTR-mediated Cl^-^ current was measured using a previously described protocol [36]. Specifically, cellular levels of cAMP were increased with forskolin (10μM) and 3-isobutyl-1-methylxanthine (IBMX, 100 μM) and the cAMP-stimulated current after apical addition of forskolin and IBMX (Isc_F&I_) was measured. 

### Statistics

Data were analyzed through calculation of group means and standard error of the mean (SEM) for each group. The analysis was performed using an unpaired t-test or ANOVA and *P*<0.05 was defined as statistically significant.

## Supporting Information

File S1 **Figure S1, Staining of sorted α6^+^/E-cad^+^ cells with β4.** Epithelial cells were isolated from a normal human lung, and surface markers α6 and E-cadherin were labelled with Alexa Fluor-568 and Alexa Fluor-647, respectively, and analyzed by FACS. Populations of α6^+^/E-cad^+^ cells were cytospinned on slides and immediately immunostained with an antibody against β4 (green). Nuclei were stained with DAPI (blue). Scale bar= 50 µm. ***Figure S2, Control for SPC antibody***. Populations of α6^+^/E-cad^+^ cells were cytospinned on slides and immediately immunostained with antibodies against SPC (red) and CC10 (green). Nuclei were stained with DAPI (blue). Scale bar= 100 µm. ***Figure S3: Additional images of de novo induction of K-5 expression in human α6^+^ cells***. Fluorescence images of a dual GFP (green) and mCherry (red)-positive colony at 4 and 7 days of culture of α6^+^ cells infected with the dual-color lentiviral reporter and co-cultured with HUVECs. Also shown are phase contrast images of colonies. Scale bar=100 µm. Note that the cluster of cells does not increase in size between days 4 and 7, which suggests that freshly isolated α6^+^ cells that were originally K-5^+^ do not undergo clonal expansion in the tested culture conditions. This is in contrast to colonies that were originally GFP-positive/mCherry-negative that later became mCherry-positive (see Figure 6B).
(PDF)

Video S1 **Time-lapse videography of clonal expansion of α6^+^ epithelial cells isolated from the distal human lung.** *This video can be viewed using QuickTime Player.
(MOV)
