# Supplementary material for: Integrin α6β4 Identifies Human Distal Lung Epithelial Progenitor Cells with Potential as a Cell-Based Therapy for Cystic Fibrosis Lung Disease
Source: PLoS One. 2013 Dec 12;8(12):e83624. doi: 10.1371/journal.pone.0083624 (PMC3861522; doi:10.1371/journal.pone.0083624)
Supplement: File S1 — Figure S1, Staining of sorted α6+/E-cad+ cells with β4. Epithelial cells were isolated from a normal human lung, and surface markers α6 and E-cadherin were labelled with Alexa Fluor-568 and Alexa Fluor-647, respectively, and analyzed by FACS. Populations of α6+/E-cad+ cells were cytospinned on slides and immediately immunostained with an antibody against β4 (green). Nuclei were stained with DAPI (blue). Scale bar= 50 µm. Figure S2, Control for SPC antibody. Populations of α6+/E-cad+ cells were cytospinned on slides and immediately immunostained with antibodies against SPC (red) and CC10 (green). Nuclei were stained with DAPI (blue). Scale bar= 100 µm. Figure S3: Additional images of de novo induction of K-5 expression in human α6+ cells. Fluorescence images of a dual GFP (green) and mCherry (red)-positive colony at 4 and 7 days of culture of α6+ cells infected with the dual-color lentiviral reporter and co-cultured with HUVECs. Also shown are phase contrast images of colonies. Scale bar=100 µm. Note that the cluster of cells does not increase in size between days 4 and 7, which suggests that freshly isolated α6+ cells that were originally K-5+ do not undergo clonal expansion in the tested culture conditions. This is in contrast to colonies that were originally GFP-positive/mCherry-negative that later became mCherry-positive (see Figure 6B). (PDF) [file pone.0083624.s001.pdf]

Supporting Information

Integrin α6β4 identifies human distal lung epithelial progenitor cells with potential as a cell-based therapy for cystic fibrosis lung disease

Xiaopeng Li, Nathan Rossen, Patrick L. Sinn, Andrew L.Hornick, Benjamin Steines, Philip H. Karp, Sarah E. Ernst, Ryan J. Adam, Thomas O. Moninger, Dana Levasseur, Joseph Zabner

Supporting Figure S1: Staining of sorted $\alpha6^+$/E-cad$^+$ cells with β4.

Supporting Figure S2: Control for SPC antibody.

Supporting Figure S3: Additional images of *de novo* induction of K-5 expression in human $\alpha6^+$ cells.

Supporting Video Image S1: Time-lapse videography of clonal expansion of $\alpha6^+$ epithelial cells isolated from the distal human lung. **This video can be viewed using QuickTime Player.*

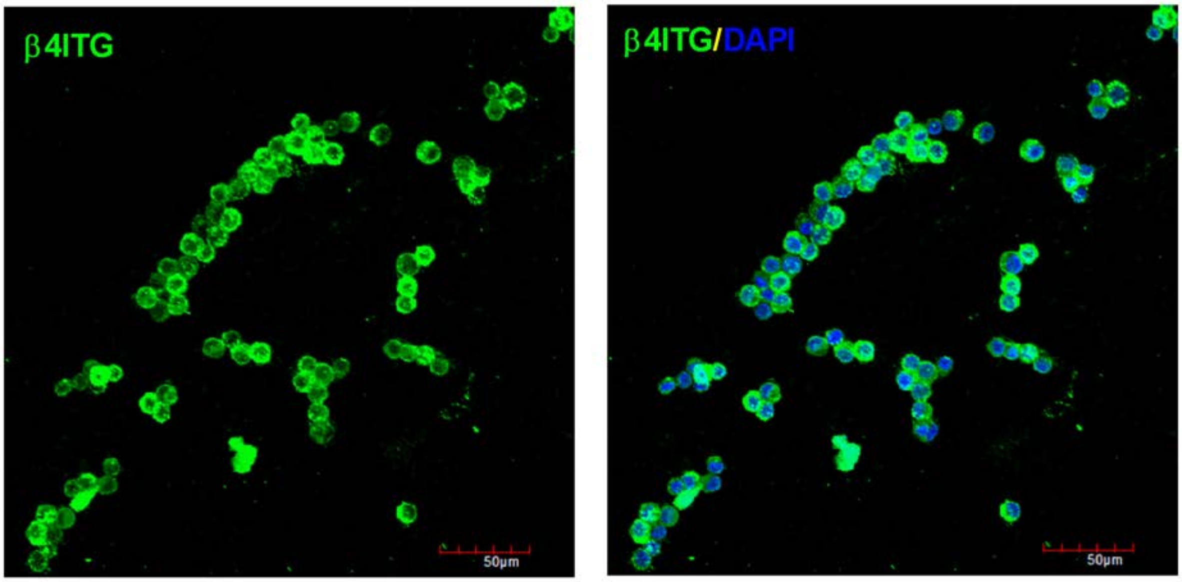

Supporting Figure S1: Staining of sorted α6^{+}/E-cad^{+} cells with β4. Epithelial cells were isolated from a normal human lung, and surface markers α6 and E-cadherin were labelled with Alexa Fluor-568 and Alexa Fluor-647, respectively, and analyzed by FACS. Populations of α6^{+}/E-cad^{+} cells were cytospinned on slides and immediately immunostained with an antibody against β4 (green). Nuclei were stained with DAPI (blue). Scale bar= 50 µm.

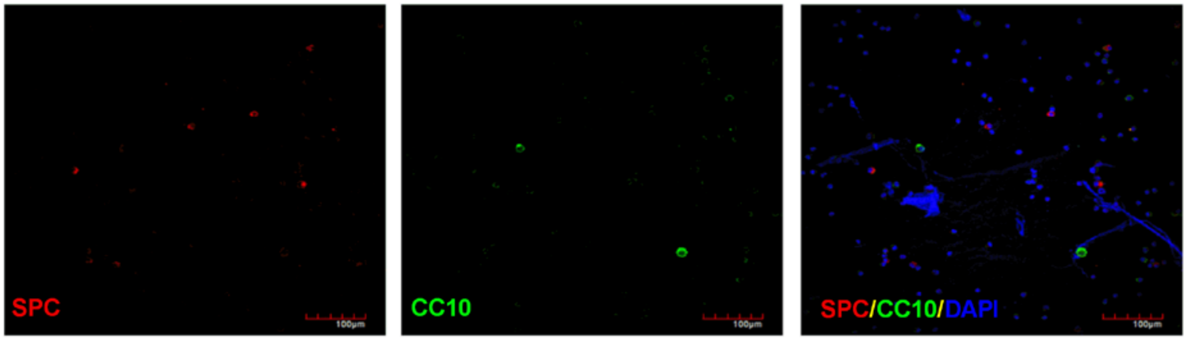

Supporting Figure S2: Control for SPC antibody. Populations of $\alpha6^+$/E-cad$^+$ cells were cytospinned on slides and immediately immunostained with antibodies against SPC (red) and CC10 (green). Nuclei were stained with DAPI (blue). Scale bar= 100 µm.

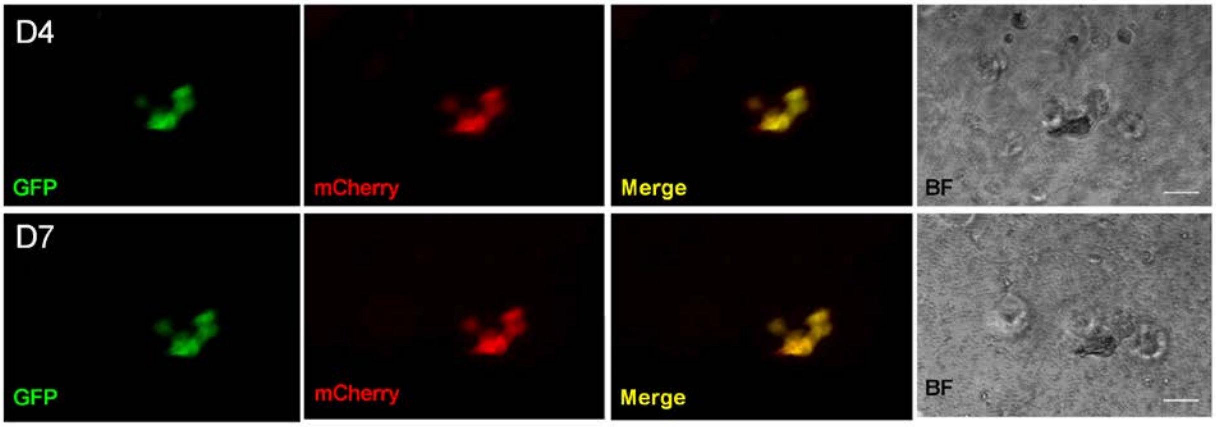

Supporting Figure S3: Additional images of *de novo* induction of K-5 expression in human α6^{+} cells. Fluorescence images of a dual GFP (green) and mCherry (red)-positive colony at 4 and 7 days of culture of α6^{+} cells infected with the dual-color lentiviral reporter and co-cultured with HUVECs. Also shown are phase contrast images of colonies. Scale bar=100 µm. Note that the cluster of cells does not increase in size between days 4 and 7, which suggests that freshly isolated α6^{+} cells that were originally K-5^{+} do not undergo clonal expansion in the tested culture conditions. This is in contrast to colonies that were originally GFP-positive/mCherry-negative that later became mCherry-positive (see Figure 6B).
